# Inhibiting ubiquitination causes an accumulation of SUMOylated newly synthesized nuclear proteins at PML bodies

**DOI:** 10.1074/jbc.RA119.009147

**Published:** 2019-07-08

**Authors:** Zhe Sha, Tamara Blyszcz, Román González-Prieto, Alfred C. O. Vertegaal, Alfred L. Goldberg

**Affiliations:** ‡ Department of Cell Biology, Harvard Medical School, Boston, Massachusetts 02115; § Department of Cell and Chemical Biology, Leiden University Medical Center, Leiden 2333 ZA, The Netherlands

**Keywords:** ubiquitin, proteasome, ubiquitylation (ubiquitination), small ubiquitin-like modifier (SUMO), sumoylation, nuclear body, promyelocytic leukemia (PML), SUMO-conjugating enzyme UBC9, TAK243, ubiquitin-activating enzyme (UAE/E1))

## Abstract

Protein ubiquitination and SUMOylation are required for the maintenance of cellular protein homeostasis, and both increase in proteotoxic conditions (*e.g.* heat shock or proteasome inhibition). However, we found that when ubiquitination was blocked in several human cell lines by inhibiting the ubiquitin-activating enzyme with TAK243, there was an unexpected, large accumulation of proteins modified by SUMO2/3 chains or SUMO1, but not by several other ubiquitin-like proteins. This buildup of SUMOylated proteins was evident within 3–4 h. It required the small ubiquitin-like modifier (SUMO)-conjugating enzyme, UBC9, and the promyelocytic leukemia protein (PML) and thus was not due to nonspecific SUMO conjugation by ubiquitination enzymes. The SUMOylated proteins accumulated predominantly bound to chromatin and were localized to PML nuclear bodies. Because blocking protein synthesis with cycloheximide prevented the buildup of SUMOylated proteins, they appeared to be newly-synthesized proteins. The proteins SUMOylated after inhibition of ubiquitination were purified and analyzed by MS. In HeLa and U2OS cells, there was a cycloheximide-sensitive increase in a similar set of SUMOylated proteins (including transcription factors and proteins involved in DNA damage repair). Surprisingly, the inhibition of ubiquitination also caused a cycloheximide-sensitive decrease in a distinct set of SUMOylated proteins (including proteins for chromosome modification and mRNA splicing). More than 80% of the SUMOylated proteins whose levels rose or fell upon inhibiting ubiquitination inhibition underwent similar cycloheximide-sensitive increases or decreases upon proteasome inhibition. Thus, when nuclear substrates of the ubiquitin–proteasome pathway are not efficiently degraded, many become SUMO-modified and accumulate in PML bodies.

## Introduction

In mammalian cells, the ubiquitin–proteasome system (UPS)^4^ (1) catalyzes degradation of most cellular proteins, including nearly all short-lived proteins and about 70% of the much more abundant long-lived proteins (2). The rapid degradation of short-lived proteins by the UPS is essential for many biological processes, and the selective hydrolysis of misfolded proteins by the UPS is critical in protein quality control. In this pathway, nuclear and cytosolic proteins are targeted for degradation by covalent attachment of chains of ubiquitin (Ub) on a lysine residue via a cascade of enzymes, including the Ub-activating enzymes (UAE, E1), Ub-conjugating enzymes (E2), and Ub ligases (E3) (3). Ubiquitinated proteins are then bound by the 26S proteasome, where they are unfolded and degraded, and the Ub molecules are released (4).

In addition to Ub, eukaryotic cells encode nearly 20 Ub-like proteins that can also be post-translationally linked to proteins and other molecules (5). The C termini of these Ub-like proteins are conjugated to substrates by a similar enzymatic cascade as Ub but by their own E1, E2, and E3s (1). The modification by the small Ub-related modifier (SUMO), termed SUMOylation, has attracted particular attention because this pathway modifies more than 1000 target proteins (6) and influences diverse cellular processes, including nuclear transport, transcription, chromatin remodeling, DNA repair, cell cycle, and ribosomal biogenesis (7). SUMOylation is essential in most organisms from yeast to mammals (8) and is frequently up-regulated in human cancers (9). Most vertebrates express three SUMO proteins, SUMO1–3, which are all found predominantly in the nucleus where they modify mostly nuclear proteins. SUMO2 and SUMO3 share 97% sequence identity and have indistinguishable functions (therefore, we refer to them here as SUMO2/3). Both share roughly 50% sequence identity with SUMO1 (10). Conjugation of all three SUMOs to substrates requires the dimeric SUMO-activating enzyme (SAE1/SAE2) and the SUMO-conjugating enzyme Ubc9, which catalyzes the covalent linkage to an ϵ-amino group of a lysine residue in the substrate, frequently within the SUMOylation consensus motif (SCM) ψK*X*E (where ψ is a large hydrophobic residue; *X* is any residue) (8). Unlike SUMO1, SUMO2/3 both contain an SCM to allow formation of Lys-11–linked poly-SUMO chains (10). Although substrates containing SCM can be SUMOylated in cell-free reactions with a high concentration of Ubc9 (11), most SUMOylation in cells needs to be accelerated by one of several SUMO ligases (E3s) (8), which also confer substrate selectivity. SUMOylation of most proteins can be readily reversed by SUMO-specific proteases (12). These proteases maintain basal SUMO conjugate levels low in cells normally, which allows cells to trigger robust modification with SUMO, especially SUMO2/3 chains upon stressful conditions (*e.g.* oxidative stress, hypoxia, osmotic stress, DNA damage, or heat shock) (5).

The biological effects of SUMOylation are mediated by the binding between SUMO and proteins containing SUMO–interaction motifs (SIMs) (13). Although SUMO binds SIMs with a weak affinity (in the low-micromolar range), in cells the SUMO–SIM interactions are frequently multivalent (by binding to proteins harboring multiple SIMs (13)) or cooperative (by simultaneous SUMOylation of multiple targets in the same protein complex (14)), resulting in formation of phase-separated protein condensates (15). The major SUMO-rich protein condensate is the promyelocytic leukemia (PML) nuclear body (PML-NB) (16), the main site in cells for protein SUMOylation. In acute promyelocytic leukemia, its main component, the PML protein, is fused with the retinoic acid receptor α, which causes disorganization of PML-NB (17, 18). By contrast, PML-NBs become more prominent when cells are exposed to oxidative stress (19), viral infection (20), proteasome inhibition (21), inflammatory stimulation by interferons or tumor necrosis factor (22), or the expression of oncogenic Ras (23). Although their exact role is still uncertain, PML-NBs and the associated SUMOylated proteins have been implicated in many nuclear processes, including transcription, DNA repair, cell cycle, apoptosis, and senescence (24). However, PML knockout mice are viable, although they have a higher incidence of tumors (25). SUMO-mediated association of PML proteins constitutes the nucleation event in PML-NB formation, and PML thus functions as a scaffold to bind both the SUMO-E2 Ubc9 and certain substrates to facilitate their SUMOylation (26, 27), which can regulate their function and promote their degradation.

Protein modifications by ubiquitination and SUMOylation have been reported to influence each other. Many lysine residues on substrates can be modified with either Ub or SUMO2/3. In fact, a systematic analysis of many thousands of SUMOylation sites revealed that 24% can also be ubiquitinated (28). Competition between SUMO and Ub conjugation for modification of the same lysine has been characterized for several proteins, including proliferating cell nuclear antigen (PCNA) (29), IκBα (30), and α-synuclein (31). In addition, SUMOylation serves as a signal for subsequent ubiquitination and degradation of many proteins (32). Chains of SUMO2/3 recruit SUMO-targeted Ub ligases (STUbLs) to promote the polyubiquitination of SUMOylated proteins, which leads to their degradation by proteasomes (32). For example, in humans, the main STUbL, RNF4, is important for triggering the degradation of PML upon arsenic trioxide treatment (32) and of DNA repair factors following homologous recombination (33, 34). STUbL-mediated ubiquitination following SUMOylation at PML-NBs also appears to play an important role in protein quality control in the nucleus. For example, in certain cell models of neurodegenerative disease, this pathway promotes the clearance of the causative nuclear proteins bearing an expanded polyglutamine sequence, including Ataxin1 and Huntingtin mutants (27).

Protein ubiquitination and SUMOylation frequently increase together under conditions such as proteasome inhibition (35) and heat shock (36), both of which cause the accumulation of many misfolded proteins. Upon proteasome inhibition, SUMOylation of mostly newly-synthesized proteins increases, whereas upon heat shock, there is greater SUMOylation of long-lived pre-existent cell proteins (35). The basis for the simultaneous increases in these two modifications is unclear. In this study, we used a selective inhibitor of the UAE, TAK243 (37, 38), to investigate the consequence of blocking ubiquitination on protein turnover. We made the unexpected finding that treating cells with TAK243, although depleting cells of ubiquitinated proteins, led to a large accumulation of proteins modified by SUMO2/3 chains. We therefore carried out detailed studies to characterize this unexpected finding, to identify the SUMOylated proteins involved, and to clarify the cellular mechanisms for their modification.

## Results

### Blocking ubiquitination causes accumulation of proteins modified with SUMO2/3 chains

**Figure 1.**
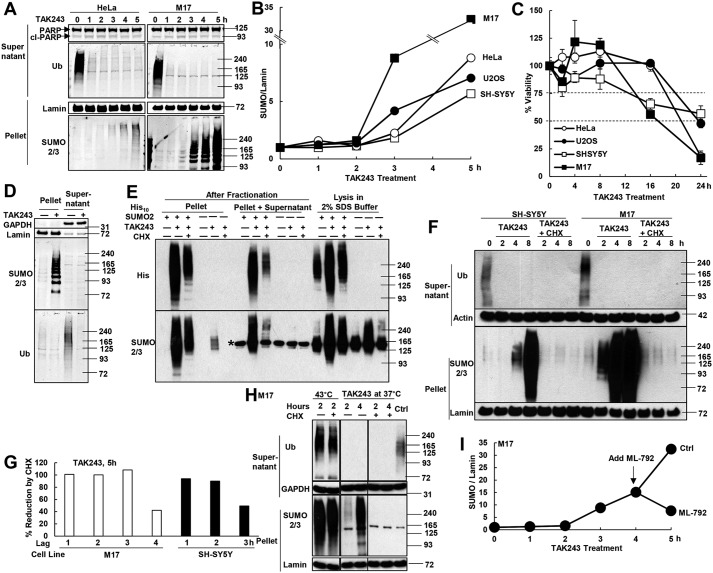
**Inhibition of ubiquitination causes accumulation of SUMOylated, newly-synthesized proteins.** *A,* after HeLa cells and M17 neuroblastoma cells were treated with 10 μm TAK243 to inhibit the UAE, proteins were fractionated by centrifugation at 10,000 × *g* for 10 min in buffer containing 1% Triton X-100. UAE inhibition depleted ubiquitinated proteins in the supernatant at 1 h and triggered the accumulation of proteins modified by SUMO2/3 chains in the pellet fraction at 3 h. There was no cleavage of PARP upon treatment for up to 5 h. *B,* four cell lines were treated with 10 μm TAK243 for 5 h. The amount of SUMO2/3-modified proteins in the pellet fraction, after normalization to lamin, was measured by Western blotting. The amount in untreated cells was set as 1. TAK243 began to cause the accumulation of SUMOylated proteins by 3 h, but not the 2-h treatments. *C,* after four cell lines were treated with 10 μm TAK243 for 2, 4, 8, 16, or 24 h, cell viability was measured by MTS assay (four individual wells for each condition). TAK243 treatment did not cause more than 25% loss of viability by 8 h and more than 50% by 16 h. *D,* M17 cells were treated with 10 μm TAK243 for 4 h and fractionated. Equal amounts of proteins were loaded for the supernatant and pellet fractions. The loading control was GAPDH for the supernatant and lamin for the pellet. Ubiquitinated proteins were predominantly in the supernatant, and SUMO2/3-modified proteins were in the pellet. *E,* HeLa cells were lysed in denaturing buffer containing 2% SDS after treatment for 4 h with 10 μm TAK243 and 100 μg/ml CHX. The denaturing lysis condition preserved more SUMOylated proteins (detected by SUMO2/3 or His antibodies) than the cell fraction procedure. However, the CHX-sensitive accumulation of SUMOylated proteins upon TAK243 treatment was still evident after lysis in 2% SDS buffer. *Asterisk* indicates a nonspecific band recognized by the SUMO2/3 antibody. *F,* SH-SY5Y and M17 cells were treated with 10 μm TAK243 and/or 100 μg/ml CHX. In the presence of CHX, there was no accumulation of SUMO2/3-modified proteins upon TAK243 treatment if measured after fractionation. *G,* SH-SY5Y and M17 cells were treated with 10 μm TAK243 for 5 h. 100 μg/ml CHX was added after 1–4 h of TAK243 treatments. After fractionation, the amount of SUMOylated proteins in the pellet was quantified by Western blotting after normalization to lamin. *H*, M17 cells were shifted to 43 °C for 2 h or treated at 37 °C with 10 μm TAK243 for 2 or 4 h. CHX (100 μg/ml) was added. Cells were fractionated and analyzed as in *A*. Unlike TAK243 treatment, shifting to 43 °C already triggered robust accumulation of SUMO2/3-modified proteins at 2 h, which was not affected by CHX. All lanes are cropped from the same blot with identical exposure. *I,* SUMOylated proteins were measured in TAK243-treated M17 cells and quantified as in *B*. Addition of the SUMO-activating enzyme SAE1 inhibitor, ML-792, at 4 h, not only prevented further accumulation of SUMOylated proteins from 4 to 5 h, but reduced pre-formed SUMO conjugates by half.

To block protein ubiquitination, we treated cells with TAK243, which prevents the transfer of activated Ub from UAE to Ub–E2s (38). After a 1-h treatment of neuroblastoma (SH-SY5Y, M17 cells), HeLa, or U2OS cells with 10 μm TAK243, ubiquitinated proteins were no longer evident (Fig. 1*A*) (data not shown). However, by 3 h, there was a large surprising accumulation of proteins modified by SUMO2/3 in all four cell lines (Fig. 1, *A* and *B*, and Fig. S1, *A–D*). Inhibition of ubiquitination thus appears to trigger a general accumulation of SUMOylated proteins in mammalian cells. This accumulation of SUMOylated proteins occurred long before marked cell death or signs of apoptosis. Treatment of all four cell lines with TAK243 for 5 h did not trigger cleavage of PARP, an indicator of apoptosis (Fig. 1*A* and data not shown), and caused less than 25% loss of viability at 8 h (measured by the MTS assay of mitochondria function) and less than 50% at 16 h (Fig. 1*C*). To test whether protein SUMOylation may be an adaptive response that enhances the viability of TAK243-treated cells, we co-treated HeLa cells with both TAK243 and ML-792, an inhibitor of the SUMO E1 SAE1 (39). This possibility seems unlikely because preventing SUMOylation with a high concentration of 20 μm ML-792 alone for 20 h reduced cell viability by 25%, but ML-792 did not alter loss of viability caused by TAK243 (Fig. S1*E*).

In these experiments (Fig. 1, *A* and *B*), we fractionated cell lysates prepared in the mild detergent (1% Triton X-100) by centrifugation (10,000 × *g* for 10 min), in order to distinguish soluble proteins and pelleted proteins, which are associated with large inclusions, chromatin, cytoskeleton, or organelles. Unlike ubiquitinated proteins, which are mostly soluble, the SUMOylated proteins were almost exclusively present in the 10,000 × *g* pellet (Fig. 1*D*). A similar enrichment of pelleted SUMOylated proteins has also been reported by us and others for cells treated with proteasome inhibitors (35, 40). Because deSUMOylation may be occurring during cell lysis and centrifugation, we also lysed cells in denaturing buffer containing 2% SDS to inactivate deSUMOylating enzymes. These conditions preserved more SUMOylated proteins, which could then be detected even in untreated cells (Fig. 1*E*). Nevertheless, using this lysis condition, TAK243 treatment still caused a marked accumulation of SUMOylated proteins.

The SUMOylated proteins that accumulate upon proteasome inhibition appeared to be mainly newly-synthesized proteins because their buildup was blocked by inhibitors of protein synthesis (35). Similarly, addition of cycloheximide (CHX) together with the TAK243 for 2–8 h almost abolished the accumulation of SUMO2/3-modified proteins in SH-SY5Y, M17 (Fig. 1*F*), HeLa, and U2OS cells (Fig. S1, *A–D*). After lysing cells in denaturing condition, as described above to prevent deSUMOylation, we could still observe a dramatic suppression of TAK243-triggered accumulation of SUMO2/3 conjugates by CHX co-treatment (Fig. 1*E*). Thus TAK243 treatment, like proteasome inhibitors, appears to lead to SUMOylation selectively of newly-synthesized proteins.

Upon TAK243 treatment, there was a time delay between the depletion of ubiquitinated proteins (at 1 h) and the accumulation of SUMOylated proteins (at 3 h) (Fig. 1, *A* and *B*). Therefore, we tested whether protein synthesis during this time delay is important for SUMOylated proteins to accumulate. M17 or SH-SY5Y neuroblastoma cells were treated with TAK243 for 5 h, and CHX was added after 1–4 h. After the addition of CHX at 3 h to M17 cells or at 2 h to SH-SY5Y cells, CHX could still completely block the accumulation of SUMOylated proteins measured at the end of the 5-h treatment (Fig. 1*G*). Therefore, new protein synthesis for 2–3 h is required for TAK243 to cause the accumulation of SUMOylated proteins.

Proteotoxic stresses such as heat shock (shift to 43 °C) also trigger the accumulation of SUMO2/3-modified proteins in the pellet fraction (35). However, unlike UAE inhibition, heat-induced accumulation of SUMOylated proteins already reached a high level at 2 h and was not blocked by CHX (Fig. 1*H*), indicating that SUMO modifies primarily pre-existent proteins upon heat shock. Finally, because cells can rapidly remove SUMO modifications by SUMO proteases of the sentrin-specific protease (SENP) family (5), we tested whether SUMOylated proteins that accumulate in TAK243-treated cells can also be deSUMOylated. During a 5-h treatment of M17 cells with TAK243, we added the SUMO E1 SAE inhibitor ML-792 at 4 h. ML-792 treatment for 1 h not only suppressed the further accumulation of SUMO2/3-modified proteins (from 15- to 32-fold in control cells), but instead reduced their level by nearly half (from 15- to 8-fold) (Fig. 1*I*).

### SUMOylation of these proteins requires Ubc9

TAK243 was reported to be a highly-specific inhibitor of the UAE (38), although it may weakly inhibit activating enzymes (E1s) for other Ub-like proteins. Because TAK243 at 10 μm caused the appearance and accumulation of SUMO-modified proteins, at this concentration TAK243 must inhibit UAE but not the SUMO-activating enzyme (SAE). Because TAK243 prevented the activation of Ub and its transfer to the various Ub–E2s, these uncharged Ub–E2s may perhaps be nonspecifically charged with SUMO or another Ub family member (Ub-like protein), whose activating enzymes (E1s) are affected by TAK243 to a much lower extent than UAE (38). Consequently, the activated Ub-like protein may be conjugated by Ub–E2 to proteins.

We therefore tested whether TAK243 treatment also elevates protein modification with other Ub-like proteins. In addition to SUMO2/3, treatment of HeLa or U2OS cells with TAK243 for 4 h increased proteins modified with SUMO1, and the conjugation of SUMO1 (like that of SUMO2/3) also was prevented by exposure to CHX (Fig. S2*A*). However, blocking ubiquitination did not increase the levels of proteins conjugated to three other Ub-like proteins Ufm1, Isg15, or Nedd8 (Fig. S2*A*). The amount of Nedd8-modified proteins actually decreased upon TAK243 treatment (Fig. S2*A*), perhaps because in the absence of ubiquitination, the major pool of Nedd8-modified proteins, the Cullin-based E3s, are deneddylated. Because TAK243 also reduced levels of Nedd8-modified proteins (41), we tested whether the SUMOylation upon TAK243 treatment is due to an inhibition of neddylation. However, inhibition of neddylation by MLN-4924 did not increase protein SUMOylation (Fig. S2*B*). Thus, the buildup of SUMOylated proteins appears to be a specific consequence of Ub conjugation defects.

**Figure 2.**
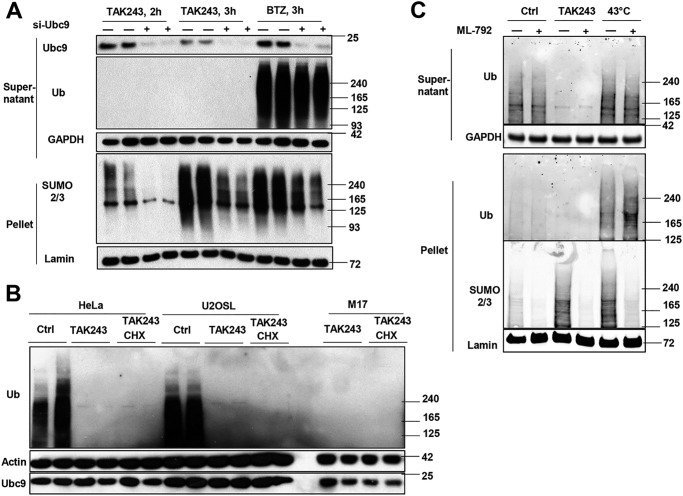
**UAE inhibition increases levels of SUMOylated proteins through SAE1 and Ubc9 but not the level of Ubc9.** *A,* knockdown of Ubc9 by siRNA dramatically reduced SUMO2/3-modified proteins in HEK293A cells treated with 10 μm TAK243 or 1 μm proteasome inhibitor BTZ. *B,* treatment of HeLa, U2OS, or M17 cells for 4 h with TAK243 (10 μm) or CHX (100 μg/ml) did not affect the level of Ubc9 in the cell lysate. *C,* treatment of M17 cells with the SAE1 inhibitor ML-792 (20 μm) abolished the SUMOylation upon treatment with TAK243 (10 μm, 4 h) or heat shock (43 °C, 2 h), but did not affect the level of ubiquitinated proteins.

To further examine the dependence on the SUMOylation machinery, we tested whether this accumulation of proteins modified by SUMO1, -2, and -3 required the SUMO-specific E2 Ubc9. We achieved a large reduction of Ubc9 by siRNA in HEK293A cells, which caused a strong decrease in proteins linked to SUMO2/3 following the blockage of ubiquitination for 2 or 3 h (Fig. 2*A*) or of protein degradation with the proteasome inhibitor bortezomib (BTZ) for 2 h. Therefore, the inhibition of ubiquitination causes a specific increase in levels of proteins modified by all three SUMO homologs via Ubc9, although the cell content of Ubc9 did not change upon treatment with TAK243 or CHX (Fig. 2*B*). We also tested the converse, whether inhibition of SUMOylation causes an accumulation of ubiquitinated proteins. Following inhibition of the SUMO-activating enzyme SAE1 with ML-792 (39), there was no change in the levels of ubiquitinated proteins (Fig. 2*C*).

### Buildup of SUMOylated proteins occurs at PML bodies and requires the PML protein

**Figure 3.**
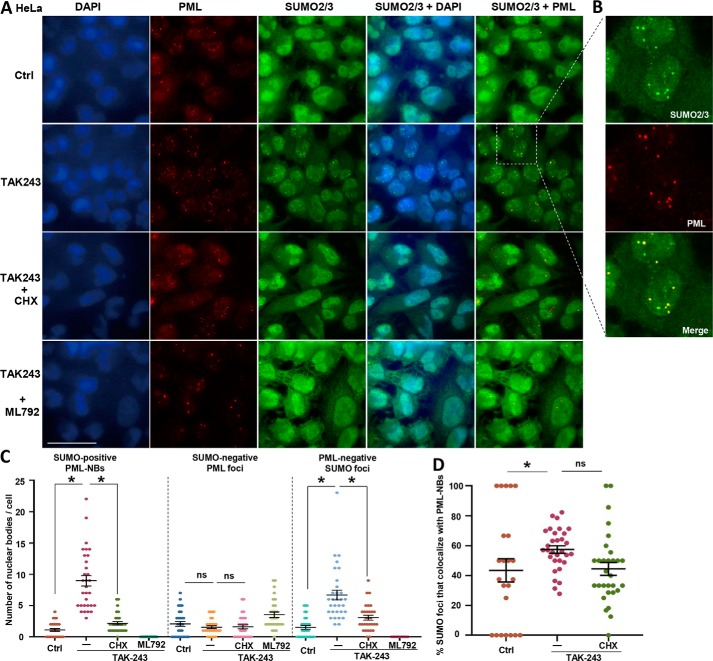
**UAE inhibition caused accumulation of SUMO2/3-modified proteins at PML-NBs.** *A* and *B,* after HeLa cells were treated for 4 h with TAK243 (10 μm), CHX (100 μg/ml), or ML-792 (20 μm), the localization of PML and SUMO2/3 was measured by immunostaining. Magnitude of the field indicated by the *dotted line box* was increased in *B* to better demonstrate the co-localization of PML and SUMO2/3 foci. The *scale bar* is 50 μm. *C,* numbers of PML-NBs (SUMO^+^, PML^+^), PML foci (SUMO^−^, PML^+^), and PML-negative SUMO foci (SUMO^+^, PML^−^) were quantified per cell. *D,* percentage of SUMO^+^ foci that co-localize with PML-NBs was also measured. TAK243 triggered the accumulation of SUMO2/3 conjugates at nuclear foci that mostly co-localize with PML. Co-treatment with CHX or ML-792 reduced the number of PML-NBs, but not SUMO-negative PML foci. 20–30 cells were quantified, and average ± S.E. were plotted. *, *p* < 0.05 statistically significant; *ns*, statistically not significant.

Most proteins modified by SUMO2/3 are found in the nucleus. However, because those SUMOylated proteins during TAK243 treatment appear to be newly synthesized, their SUMOylation may occur in the cytoplasm. We therefore immunostained with SUMO2/3 antibody in HeLa cells and HeLa cells expressing His_10_–SUMO2 after incubation with TAK243 for 4 h. This treatment caused a dramatic increase in SUMO2/3-positive nuclear foci, from 3 per cell to 16 per cell in HeLa (Fig. 3, *A–C*) and from 0.3 per cell to 13 per cell in the HeLa/His_10_–SUMO2 cells (Fig. S3, *A* and *B*). Simultaneously, TAK243 also increased the number of nuclear foci containing the PML protein from 4 per cell to 11 per cell in HeLa cells (Fig. 3, *A–C*) and from 5 per cell to 10 per cell in HeLa/His_10_–SUMO2 (Fig. S3, *A* and *B*). In addition, this treatment caused a very large increase in the average number of PML-NBs, *i.e.* foci that stain positive for both SUMO2/3 and PML (from 1 per cell to 9 per cell in HeLa cells (Fig. 3, *A–C*) and from 0.1 per cell to 9 per cell in HeLa/His_10_–SUMO2 cells (Fig. S3, *A* and *B*)). By contrast, the number of PML foci lacking SUMO2/3 seemed to slightly decrease in HeLa cells (Fig. 3, *A–C*) and decreased from 5 per cell to 1 per cell in HeLa/His_10_–SUMO2 cells (Fig. S3, *A* and *B*). Furthermore, all these changes in PML-NBs and their content of SUMOylated proteins were inhibited by the presence of CHX (Fig. 3, *A–C*, and Fig. S3, *A* and *B*).

Upon TAK243 treatment, 58% SUMO2/3-positive foci co-localized with PML-NBs in HeLa cells (Fig. 3*D*) and 72% in HeLa/His_10_–SUMO2 cells (Fig. S3*C*), whereas no SUMO2/3-positive foci were observed outside the nuclei. To confirm that these SUMO2/3-positive foci were formed by protein SUMOylation, we treated cells with both TAK243 and ML-792, which blocked formation of these foci (Fig. 3, *A* and *B*, and Fig. S3*A*). Therefore, upon UAE inhibition, a pool of newly-synthesized proteins are modified by SUMO2/3 and accumulated mainly in PML-NBs. To confirm that UAE inhibition triggers the buildup of SUMOylated proteins mainly in the nucleus, we treated M17 cells with TAK243 for 4 h and then fractionated the cell lysates into cytoplasmic, nuclear, and chromatin-bound fractions. Most SUMOylated proteins were recovered in the chromatin-bound fraction (Fig. S3*D*). Because PML-NBs make tight contact with the chromatin (42), often in transcriptionally-active genomic regions (43), this association of SUMOylated proteins with both chromatin and PML-NBs is not surprising.

**Figure 4.**
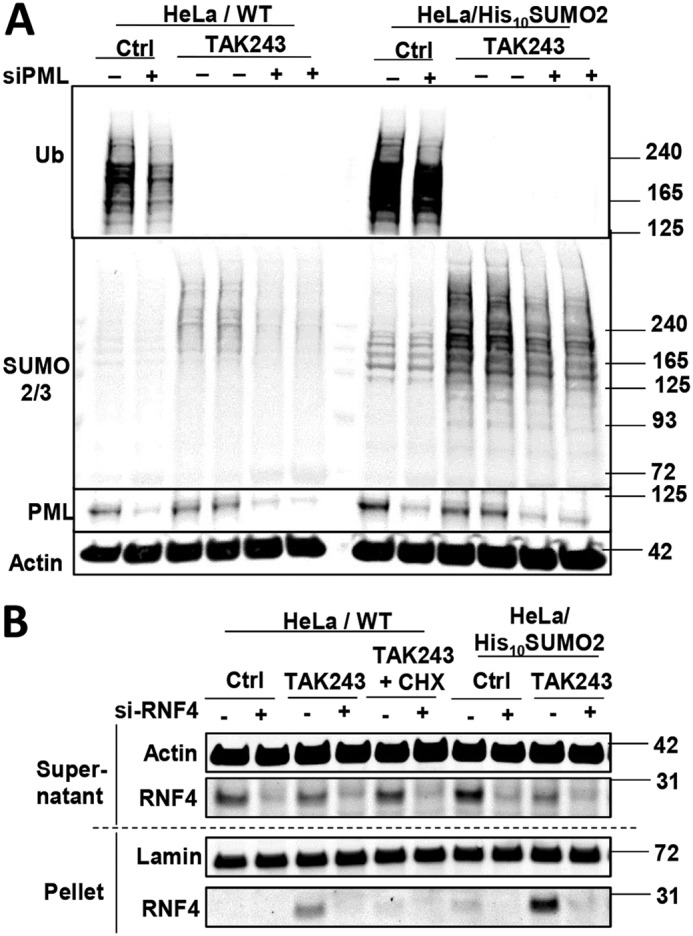
**Buildup of SUMOylated proteins upon UAE inhibition is promoted by PML.** *A,* knockdown of PML by siRNA in HeLa cells (parental and His_10_–SUMO2 cells) strongly reduced the amount of SUMO2/3-modified proteins in whole lysate of cells treated with TAK243 for 4 h. *B,* distribution of RNF4 between the supernatant and pellet fractions was determined in HeLa cells (parental and His_10_–SUMO2 cells). To confirm the identity of the RNF4 band, we used siRNA to knock it down by 70%. RNF4 was barely detectable in the pellet fractions normally, but accumulates upon TAK243 treatment in a CHX-sensitive manner.

Because most SUMO2/3 conjugates co-localized with PML-NBs, we also investigated whether the PML protein, which may facilitate the SUMOylation of aggregation-prone proteins (27), also is important upon UAE inhibition. In both HeLa cell lines, knockdown of PML strongly reduced the amount of SUMOylated proteins following TAK243 treatment (Fig. 4*A*). Even though the PML bodies increased and were more prominent, the total cellular level of PML did not change after the treatment with TAK243 (Fig. 4*A*). Thus, upon UAE inhibition, SUMOylation of nuclear proteins seemed to increase sharply in PML-NBs and required the PML protein and Ubc9.

We next tested whether the buildup of SUMOylated proteins required a single SUMO ligase. Knockdown of each of the four SUMO ligases, PIAS1, -2, -3, or -4, did not reduce the amount of SUMOylated proteins (Fig. S4*A*) after E1 inhibition. Therefore, this SUMOylation is not dependent on any single SUMO ligase of the PIAS family. These enzymes are known to have overlapping roles (44). Thus, it will be necessary to knock down multiple PIAS family members simultaneously to rigorously test their involvement, but this is technically very challenging.

### Do SUMOylated proteins accumulate because of defects in SUMO-dependent degradation?

Many SUMOylated proteins are subsequently ubiquitinated at PML-NBs by STUbLs, RNF4 or RNF111, which leads to their degradation by the 26S proteasome (32, 45). Therefore, it seemed possible that UAE inhibition may cause the accumulation of SUMOylated proteins by blocking their ubiquitination by a STUbL. Because RNF4 ubiquitinates SUMOylated proteins mainly at PML-NBs, we tested whether RNF4 is enriched at PML-NBs with or without UAE inhibition. HeLa cell lysates were fractionated as in Fig. 1*A*, and the level of RNF4 in the 10,000 × *g* pellet (which contains all PML-NBs) was assayed by Western blotting. In untreated cells, RNF4 was barely detectable in the pellet, but increased dramatically upon UAE inhibition in a CHX-sensitive manner (Fig. 4*B*), whereas the total level of RNF4 in cells was not affected by TAK243 or CHX. Therefore, RNF4 presumably builds up on PML-NBs when their SUMOylated substrates accumulate there. However, knockdown of RNF4 with siRNA by more than 70% in HeLa cells caused only a small (20–40%) buildup of SUMO-modified proteins (data not shown), in contrast to the very large increases (6–9-fold) seen upon TAK243 treatment for 4 h. It thus appears that most nuclear proteins are not primarily ubiquitinated by RNF4, although RNF4 may serve as a back-up E3 to ubiquitinate these proteins if they accumulate as SUMOylated proteins at PML-NBs. It remains possible that another unidentified STUbL is continuously ubiquitinating nuclear proteins in a SUMO-dependent manner, although the knockdown of another STUbL, RNF111, failed to cause an accumulation of SUMO conjugates (data not shown).

### Identification of SUMO2/3-modified proteins in cells treated with TAK243 by LC-MS/MS

**Figure 5.**
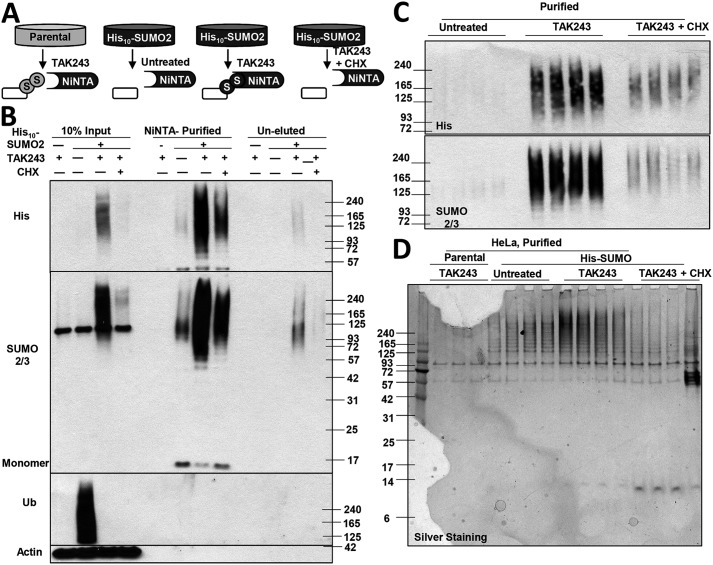
**Purification of His_10_–SUMO2-modified proteins from HeLa cells treated with TAK243.** *A,* scheme of the plan for purification of His_10_–SUMO2-modified proteins following UAE inhibition. Cells expressing His_10_–SUMO2 were treated with TAK243 (10 μm) for 4 h, and then His_10_–SUMO2-modified proteins were purified by Ni-NTA–agarose under denaturing conditions. As controls, His_10_–SUMO2-modified proteins were purified from untreated cells or cells co-treated with TAK243 and CHX (100 μg/ml), and a mock purification was performed on parental cells treated with TAK243. Four individual purifications were performed for each condition. *B,* purification of His_10_–SUMO2-modified proteins. In proteins purified from TAK243-treated cells, there were much more SUMO2/3-modified proteins and less SUMO monomer than in proteins compared with purified from untreated cells or cells treated with both TAK243 and CHX. Proteins bound to Ni-NTA–agarose were almost completely eluted. *C* and *D,* amount of proteins modified by His_10_–SUMO2 (*C*) or total proteins (assayed by silver staining, *D*) were measured from all four replicates of purified proteins. The enrichment of SUMOylated proteins and total proteins in Ni-NTA–purified samples from cells treated with TAK243 was confirmed in all four replicates.

To identify proteins modified by SUMO2/3, we used nanoflow LC-tandem MS approach (nano LC-MS/MS) (46). To facilitate purification of the SUMOylated proteins by Ni-NTA–agarose, we used HeLa and U2OS cells stably expressing His_10_–SUMO2 that we constructed previously. Upon UAE inhibition, His_10_–SUMO2, like endogenous SUMO2, also modifies proteins in a CHX-sensitive manner (Fig. S1, *A–D*). As controls, the SUMOylated proteins were purified from the His_10_–SUMO2 cells that were not exposed to TAK243 or were treated for 4 h with both TAK243 and CHX (100 μg/ml), as well as from TAK243-treated HeLa cells not expressing His_10_–SUMO2 (Fig. 5*A*). As expected, from the cells treated with TAK243, the Ni-NTA method yielded increased levels of SUMO2/3-modified proteins (Fig. 5, *B* and *C*), which was also shown by silver staining (Fig. 5*D*), but did not from the cells also treated with CHX. This increase in SUMOylated proteins in TAK243-treated cells caused a simultaneous reduction in the levels of unconjugated His_10_–SUMO2 monomer isolated with the Ni-NTA columns (Fig. 5*B*). SUMOylated proteins were also successfully isolated by this method from U2OS cells (Fig. S5). The purified His_10_–SUMO2-containing proteins were then digested with trypsin and analyzed by nano-LC–MS/MS in a label-free approach (46). Heatmap analysis of the Z-score of the label-free quantification values of the identified proteins revealed a very-high reproducibility among experimental replicates (Fig. S6). Results are summarized in Table S1.

### Inhibiting ubiquitination caused increases or decreases in the levels of different SUMO2-modified proteins

**Figure 6.**
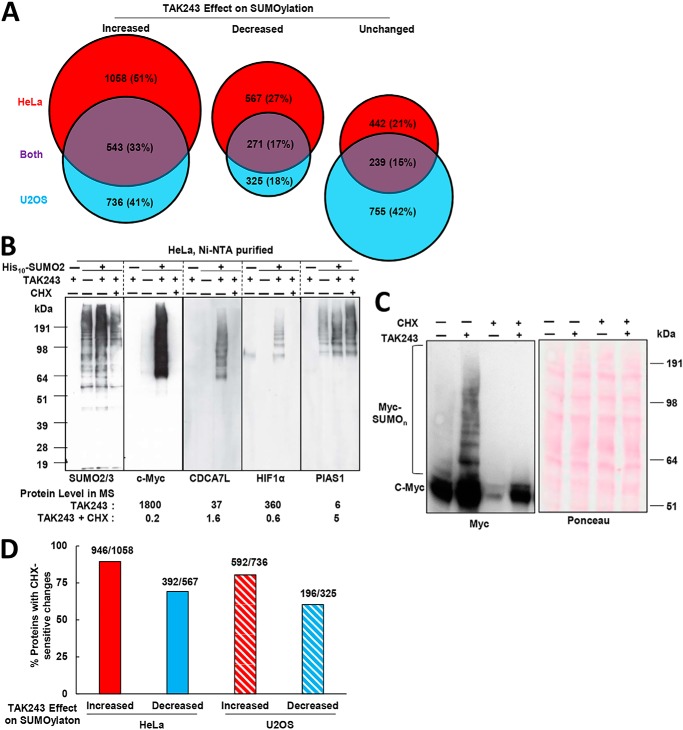
**Identification and validation of SUMO2/3-modified proteins whose levels increased after UAE inhibition.** *A,* numbers of SUMO2-target proteins whose SUMOylated species, according to MS/MS LFQ, were significantly increased, decreased, or unchanged in HeLa and/or U2OS cells. Please refer to Table S1 for the complete dataset. *B,* findings by MS were validated by Western blotting. HeLa cells stably expressing His_10_–SUMO2 were treated with 10 μm TAK243 and/or 100 μg/ml CHX for 4 h, and parental cells were treated with 10 μm TAK243 for 4 h. After His_10_–SUMO2-modified proteins were purified by Ni-NTA, the levels of SUMO2/3, c-Myc, CDCA7AL, HIF1α, and PIAS1 were measured. Changes in the level of His_10_–SUMO2-modified proteins found by MS, described at the *bottom* of the figure, match the SUMOylated protein level found by Western blotting. *C,* amounts of Myc and SUMO2/3-modified Myc were measured in lysates of HeLa cells after treatment with TAK243 (10 μm) and/or CHX (100 μg/ml). The total levels of unmodified and SUMO2/3-modified Myc were dramatically increased upon UAE inhibition but reduced upon CHX co-treatment. *D,* among SUMO2-target proteins whose SUMOylated species are significantly increased or decreased according to LC/MS/MS LFQ as described in *A*, the co-treatment with CHX negated most of these changes. Proteins with CHX-sensitive changes are ones whose SUMOylated species differ significantly between samples treated with TAK243 and samples co-treated with TAK243 and CHX.

Proteins whose LFQ levels are significantly higher in His_10_–SUMO2 cells than in parental cells were defined as SUMO2 targets. We identified 2067 SUMO2 targets in HeLa cells and 1816 in U2OS cells, among which 1636 are SUMO targets in both cell lines (Table S1 and Fig. 6*A*). After treatment with TAK243 for 4 h, the levels of 1058 SUMOylated proteins (51%) in HeLa cells and 736 (41%) in U2OS cells were increased, among which 543 (33%) increased in both cell types (Fig. 6*A*). The MS analysis indicated greater amounts of a number of SUMOylated proteins with important regulatory functions, such as c-Myc, CDCA7L, HIF1α, and PIAS1. To confirm the increases in these SUMOylated proteins, we purified His_10_–SUMO2-modified proteins from HeLa cells and found by Western blotting that the SUMOylated forms of these four proteins were elevated upon TAK243 treatment (Fig. 6*B*). Even in HeLa cell lysates the increased levels of SUMOylated c-Myc were evident following UAE inhibition, but not if CHX was also present (Fig. 6*C*).

**Figure 7.**
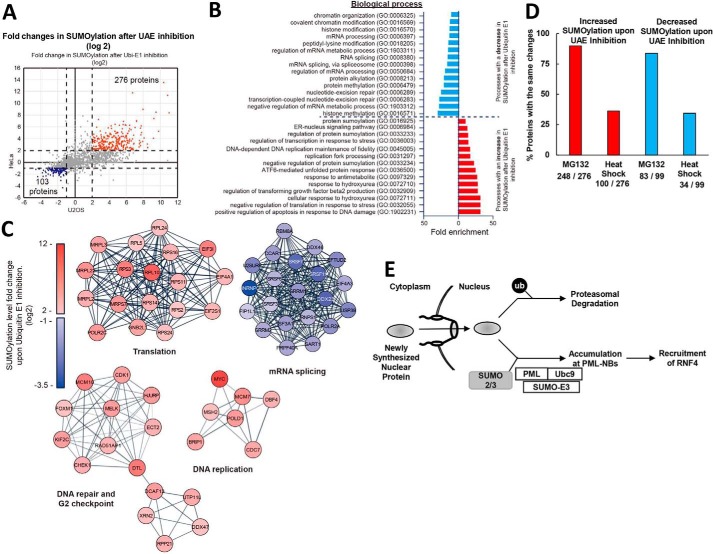
**UAE inhibition increases the amounts of SUMOylated nuclear proteins that fail to be degraded by the ubiquitin–proteasome system, and most are not ubiquitinated by RNF4.** *A,* effect of UAE inhibition on levels of SUMOylated proteins. Scatter plot depicting average LFQ difference (log_2_) between TAK243-treated and control samples for HeLa and U2OS cell lines. *Dashed lines* establish the threshold for highly-dynamic proteins upon UAE inhibition. The levels of SUMOylated species of 276 proteins were increased 4-fold or more (log_2_ value >2) in both HeLa and U2OS cells (*red*), and the levels of SUMOylated species of 103 proteins were decreased 2-fold or more (log_2_ value < −1) in both lines (*blue*). *B,* Gene Ontology (biological process) of proteins identified in *A*, whose SUMOylated species increased at least 4-fold (*red*) or decreased at least 2-fold (*blue*) upon TAK243 treatment in both lines. Please refer to Table S2 for the complete Gene Ontology analysis. *C,* specific gene clusters derived from the STRING analysis as described in Fig. S7*A*. *D,* overlap of proteins with increased or decreased SUMOylation upon TAK243 treatment (described in *A*) with proteins identified by our published MS analyses (28, 48) to undergo the same changes upon MG132 treatment or heat shock in HeLa and U2OS cells. Among the 276 proteins whose SUMOylated species increased by 4-fold or more (described in *A*), 248 (90%) also increased upon MG132 treatment (Table S3), but only 100 (36%) also increased upon heat shock (Table S5). Among the 99 proteins whose SUMOylated species decreased by 2-fold or more (described in *A*), 83 (84%) also decreased upon MG132 treatment (Table S4), but only 34 (34%) also decreased upon heat shock (Table S5). *E,* our model explaining how cells accumulate SUMOylated proteins upon the inhibition of ubiquitination, and the consequence of this modification.

Surprisingly, in response to the inhibition of ubiquitination, the levels of 567 SUMOylated proteins were reduced in HeLa cells (27%) and 325 (18%) in U2OS cells, including 271 (17%) proteins whose levels decreased in both cells (Fig. 6*A*). Furthermore, the amount of SUMOylated species of a subset of proteins did not change upon the inhibition of ubiquitination (442 (21%) in HeLa cells, 755 (42%) in U2OS cells, and 239 (15%) in both (Fig. 6*A*)). Addition of CHX together with TAK243 blocked the increase in 89% of the SUMOylated proteins (946 of 1058) in HeLa cells and 80% (592 of 736) of those in U2OS cells (Fig. 6*D*). We verified by Western blotting that although the increase in SUMOylated c-Myc, CDCA7L, and HIF1α was blocked by CHX, that of PIAS1 was not (Fig. 6*B*). Interestingly, upon UAE inhibition, CHX also prevented the decrease in 69% of the SUMOylated proteins (392 of 567) in HeLa cells and 60% of the proteins (196 of 325) in U2OS cells (Fig. 6*D*). Very similar effects of TAK243 on SUMOylation of individual protein were obtained in HeLa and U2OS cells (Fig. 7*A*). These MS studies confirmed that UAE inhibition caused a global shift of the SUMO2-modified proteins, including an increase in many newly-synthesized proteins but also a decrease in SUMOylation of many others, perhaps due to the limited pool of free SUMO.

To study the common features of the SUMOylated proteins that either increased or decreased in a highly dynamic manner following UAE inhibition, we selected 276 modified proteins, which increased at least 4-fold (log_2_ value >2)) in both cell lines, and 103 proteins, which decreased at least 2-fold (log_2_ value < −1) in both cell lines (Fig. 7*A*). Using gene ontology enrichment of cellular processes (47), we found, as anticipated, that upon UAE inhibition there was greater content of SUMOylated proteins that catalyze SUMOylation (three gene ontology categories of biological processes) (Fig. 7*B* and Table S2), but also of SUMOylated proteins involved in transcription (three categories), DNA damage response (three categories), DNA replication (two categories), and various other cellular stress responses (five categories), including endoplasmic reticulum (ER) stress (two categories) (Fig. 7*B*). Interestingly, UAE inhibition has been reported to strongly activate ER stress (38), and all three major transcription factors involved in this response (ATF4, ATF6, and XBP1) were among the 10% proteins with the greatest increase in the amount of SUMOylated protein upon UAE inhibition in both HeLa and U2OS cells (Table S1). Because their accumulation was blocked by CHX, it is likely that some of these SUMOylated proteins were synthesized to compensate for the inhibition of ubiquitination, which must prevent the degradation of misfolded ER proteins by ERAD and cause this buildup in the ER. In contrast, UAE inhibition decreased SUMOylation of proteins involved in chromatin modification, especially histone methylation, mRNA processing such as splicing, and nucleotide–excision repair (Fig. 7*B*).

We also performed String analysis to identify functional protein association networks (Fig. S7*A*). Further analysis to identify the highly-interconnected clusters within the STRING analysis identified a DNA repair and G_2_ checkpoint, a DNA replication, and translation clusters enriched in SUMOylation. However, an mRNA-splicing cluster had decreased SUMOylation (Fig. 7*C*). The functions of these networks are consistent with cellular processes found by gene ontology enrichment analysis (Fig. 7*B*). Curiously, the String analysis of proteins with greater SUMOylation also included a network of translation initiation factors, ribosomal proteins, and mitochondrial ribosomal proteins (Fig. 7*C*), which are not normally present in the nucleus, although some may be produced in the nucleolus. Therefore, UAE inhibition may also increase the levels of these SUMOylated cytoplasmic or organelle proteins, even though the majority of SUMOylated proteins accumulate in the nucleus.

### UAE inhibition causes a buildup of SUMOylated proteins by preventing their degradation

Most of the SUMOylated proteins that were isolated following UAE inhibition were associated with PML-NBs as well as chromatin (Fig. 3 and Fig. S3). To determine whether these proteins are normally nuclear constituents or perhaps were targeted to the nucleus when their ubiquitination was inhibited, we analyzed their known subcellular localizations. Among the 276 proteins, whose SUMOylation increased at least 4-fold in both HeLa and U2OS cells and are known SUMO2 targets (as described in Fig. 7*A*), 230 (83%) are assigned by gene ontology enrichment analysis to be nuclear components (data not shown).

The simplest mechanism by which UAE inhibition may increase the levels of these SUMO2/3-modified proteins would be by blocking their degradation by proteasomes, although other mechanisms are also possible. For example, preventing conjugation of Ub to certain lysine residues may allow them to be SUMOylated. We therefore tested whether UAE inhibition leads to buildup of a similar set of SUMOylated proteins as accumulate upon inhibition of the proteasome. Hendriks *et al.* (28, 48) identified the SUMOylated proteins that accumulate after treatment of HeLa and U2OS cells with the proteasome inhibitor MG132. Among the 276 SUMOylated species that increased more than 4-fold upon TAK243 treatment of both HeLa and U2OS cells (Fig. 7*A*), 248 (90%) also increased upon MG132 treatment (Fig. 7*D* and Table S3). In addition, among the 103 SUMOylated proteins (corresponding to 99 genes) that decreased more than 2-fold upon TAK243 treatment of both HeLa and U2OS cells (Fig. 7*A*), 83 (84%) also decreased upon MG132 treatment (Fig. 7*D* and Table S4). The accumulation of a very similar, probably identical, set of SUMOylated proteins following inhibition of proteasomes or ubiquitination indicates that UAE inhibition increases the level of SUMOylated proteins by blocking their degradation by the UPS.

Among the 276 most highly-SUMOylated proteins identified by MS, only 12% (32/276) are known RNF4 substrates, which were identified using TULIP methodology (targets for ubiquitin ligases identified by proteomics) (49), and only 4% (12/276) were enriched in cells when RNF4 was knocked down (Table S3) (49). Accordingly, we observed only a very small buildup of SUMO2/3-modified proteins when we knocked down RNF4 (data not shown). Thus, UAE inhibition mainly causes the accumulation of proteins that are normally not ubiquitinated by RNF4.

Finally, because exposure to heat shock has also been reported to cause similar changes in the amounts of SUMOylated proteins as occurs after proteasome inhibition (35), we compared the spectrum of proteins that accumulate or decreased SUMO2-conjugated species following heat shock (35) and TAK243 treatment. However, among 276 SUMOylated proteins that increased most upon TAK243 treatment, only 100 (36%) also accumulated upon heat shock (Fig. 7*D* and Table S5). Furthermore, among these 99 genes corresponding to 103 SUMOylated proteins that displayed reduced SUMOylation in response to TAK243, only 34 (34%) also decreased upon heat shock (Fig. 7*D* and Table S5). Thus, although changes in the levels of SUMOylated proteins are very similar upon blocking ubiquitination or proteasomes (84–90%), most of the SUMOylated species that rise and fall in heat shock are different, although heat shock does affect a large fraction (35%) of the same SUMOylated proteins that rise or fall after inhibition of the UPS.

## Discussion

Because of the central role of ubiquitination in regulating multiple essential processes, and protein quality control, blocking Ub activation has major deleterious effects on cells and eventually causes apoptosis. Consequently, TAK243 and related molecules are under active investigation and clinical trials as anti-cancer agents (37, 38). This study has described a new and surprising response to blocking ubiquitination, a buildup of many SUMOylated proteins that is evident 3 h after the depletion of ubiquitinated proteins. Normally, a large pool of newly-synthesized nuclear proteins are rapidly ubiquitinated and degraded by the proteasome after they have been transported into the nucleus (Fig. 7*E*). Addition of TAK243 causes the disappearance of Ub conjugates within 1 h and a buildup of SUMOylated proteins another 2 h later. This effect long precedes the death of these cells, which is not evident for at least 16 h. Thus, the accumulation of SUMOylated proteins and the formation of PML-NB (Fig. 7*E*) are not consequences of cells dying, although this phenomenon may somehow contribute to cell dysfunction and the eventual apoptosis. In contrast, the segregation of some of these nondegraded proteins into PML-NBs may help protect the cell against some of the harmful consequences of the blockage of ubiquitination. Although this increase in SUMOylated proteins may result from an increased rate of SUMOylation and may represent a specific mechanism to defend against the buildup of nondegraded proteins in the nucleus, there are alternative simpler explanations for their accumulation, as discussed below. Also, we could not obtain evidence for an important protective role for the accumulation of SUMOylated proteins. For example, the inhibition of SUMOylation did not enhance the cell killing by TAK243.

The physiological consequences of this buildup of SUMOylated species in the 10,000 × *g* pellet in association with chromatin is unclear at present. In addition to targeting many nuclear proteins for proteasomal degradation, attachment of SUMO molecules to a protein can alter its function. In fact, many of the transcription factors, which accumulate as SUMOylated proteins, are ones whose activities are altered by SUMO1 modification, including ATF6 (50), HSF1 (51), HSF2 (52), SMAD3 (53), SMAD4 (54, 55), and FoxM1 (56), whereas the activities of XBP1 (57) and FoxM1 (58) are altered by linkage to SUMO2/3. However, it is impossible to predict whether the buildup of these transcription factors in association with chromatin and PML-NB leads to an overall increase or decrease of transcription, and even less is known how the activities of other nuclear proteins are affected by their SUMOylation.

Because cells contain hundreds of Ub ligases and dozens of Ub–E2s with distinct properties, we had initially anticipated that one of the uncharged Ub–E2s might become linked by the SUMO E1 to a SUMO molecule leading to inappropriate SUMOylation of proteins. However, the present observations clearly indicate a highly specific response that requires Ubc9 and the PML-NB, the primary cellular site where SUMOylated proteins accumulate. Also the great majority of the SUMOylated proteins were newly synthesized nuclear proteins with diverse functions, and not cytoplasmic proteins that might be transported into the nucleus after UAE inhibition.

Blocking UAE activity thus seems to expose (or perhaps enhance) a process occurring normally to many nuclear proteins. Therefore, the simplest and most likely explanation of these phenomena is that the UAE inhibitor simply blocks the ubiquitin-dependent degradation of such SUMOylated proteins causing their accumulation on the PML-NB (Fig. 7*E*). Accordingly, the SUMOylated proteins that accumulate upon UAE inhibition are almost identical to those that increase upon proteasome inhibition.

These findings would imply that the UAE inhibitor causes their buildup by preventing the action of one or more STUbLs (32, 49). However, down-regulation of the major STUbL, RNF4, caused a much weaker increase in the levels of SUMOylated proteins (data not shown) than occurs upon UAE inhibition. Among the proteins that accumulate, only a very small but important fraction is known to be RNF4 substrates. These overlapping proteins include the SUMO E3s (ligases), which accumulate in their SUMOylated form upon TAK243 treatment. RNF4 accumulated to much higher amounts in the PML fraction after TAK243 treatment, presumably because it binds to the SUMOylated proteins that are most abundant there. These data are consistent with a model in which RNF4 functions as a brake on SUMOylation by targeting for degradation the active set of SUMO ligases that autoSUMOylate. Therefore, RNF4 may play a more important role when its SUMOylated substrates accumulate at PML-NBs to high levels, but normally these proteins may be ubiquitinated by another STUbL such as Hei10 (59) or some other E3s. Unfortunately, because TAK243 is an irreversible inhibitor that covalently modifies UAE (38), it is not possible to re-activate ubiquitination in TAK243-treated cells to study the Ub ligases that may act on these SUMOylated proteins.

Although the blockage of ubiquitination and degradation can explain simply the accumulation of many SUMOylated proteins, it cannot by itself also account for the decrease in many others. These two opposite responses must be linked somehow because addition of CHX not only prevented the increase in SUMOylated proteins, but also blocked the decrease in the other SUMOylated proteins. Possibly, the former group are SUMOylated first, and as they accumulate the supply of activated SUMO–Ubc9 decreases and limits the SUMOylation of the latter group. In fact, a decrease in free SUMO levels was observed under these conditions (Fig. 5*B*). Alternatively, these two groups of SUMO-modified proteins may differ in susceptibility to a specific SUMO protease. For example, accumulation at PML-NBs might protect SUMO-modified proteins from undergoing deSUMOylation by some SENPs (60). These two groups may also differ in function. In fact, our gene ontology enrichment analysis suggested that there were increased levels of SUMO-modified nuclear proteins involved in transcription, DNA damage responses, and DNA repair but reduced levels of SUMOylated proteins that catalyze DNA modification and mRNA splicing. These findings on SUMO modification may be analogous to situations where the supply of Ub is low and limits further degradation; then, the levels of ubiquitinated histones decrease through deubiquitination apparently to increase the levels of free Ub (61).

As noted above, the SUMO-modified proteins that increased upon TAK243 treatment were very similar (over 90% identical) to the set of proteins that increased upon proteasome inhibition. In both cases, the SUMOylated proteins that accumulated in the 10,000 × *g* pellet were associated with chromatin (Fig. S3*D*) and led to increased formation of PML-NBs (35). Also, both types of inhibitors decreased very similarly the levels of another set of SUMOylated proteins (84% of which were identical), and the inhibition of protein synthesis prevented both responses (35). Thus, it is very likely that their accumulation in the PML-NB results simply from blocking their degradation by the UPS (Fig. 7*E*). The one obvious difference between the proteins that accumulate with these two treatments is that the proteasome inhibitor should cause accumulation of these same proteins in a ubiquitinated as well as SUMOylated form, unlike the treatment with TAK243.

Interestingly, UAE inhibition increased the SUMOylation of many SUMO E3s, including PIAS1–4 (13), ZNF451 (62), TOPORS (63), and KIAA1586 (64), and some Ub ligases of the tripartite motif (TRIM) superfamily (*e.g.* TRIM27), which may also be able to catalyze Ubc9-mediated SUMOylation (65). This result is consistent with our previous finding that active pools of SUMO E3 ligases, marked by auto-SUMOylation, are targeted for degradation by the STUbL RNF4 (49). Blocking ubiquitination by UAE inhibition would thus interfere with STUbL-mediated elimination of active SUMO E3 ligases, which may possibly explain the observed increase in SUMOylation levels upon UAE inhibition.

Our efforts to identify a single SUMO ligase catalyzing this response have proven unsuccessful. A knockdown of the major SUMO ligases PIAS1–4 did not prevent this response, either because these enzymes act on distinct substrates, and all contribute to small degrees, or because they act in a redundant manner. Evidence for the cooperativity of different PIAS family members has been provided previously (44). The PML protein, which is essential for the accumulation of these proteins at the PML-NB and for the enlargement of these structures, may also facilitate SUMOylation and serve as an essential scaffold, but there is no clear evidence that it has *bona fide* ligase activity. In addition to binding SUMOylated proteins, PML has been reported to bind preferentially proteins with unstructured regions (27), and many of these nuclear substrates (*e.g.* transcription factors) are intrinsically unstructured proteins bearing such domains (66).

The time lag of 2–3 h between the complete blockage of Ub conjugation and the accumulation of SUMOylated proteins in the PML-NB is an intriguing feature of this response that will be important to understand. Most of these SUMOylated proteins (*e.g.* transcription factors) are short-lived proteins whose levels are maintained low normally due to their rapid hydrolysis. Therefore, after ubiquitination or proteasome inhibition, several hours maybe necessary for their continued production until sufficient amounts of these short-lived SUMO2 target proteins are present to bind and to expand the PML-NB. Also, during the several hours in the absence of nuclear proteolysis, a significant fraction of the short-lived proteins may become unfolded or damaged, which also favors association with the PML protein. A similar process is occurring in the cytoplasm, where a significant fraction (10–30%) of newly-synthesized proteins is rapidly degraded, perhaps because of a failure in folding (67). Furthermore, SUMOylation in the PML-NB is a highly cooperative process because of the ability of SUMOylated proteins to recruit SUMOylation enzymes, which contain SUMO-interaction motifs and modify more proteins that come into proximity (6, 13). Consistent with a cooperative response to substrate buildup, the accumulation of SUMO conjugates during TAK243 treatment appeared to accelerate rapidly after a 2–3-h lag time in all four cell lines studied. However, it is also possible that the lag time and requirement for protein synthesis arise because the formation of SUMOylated proteins may require the synthesis of some novel SUMOylation enzymes, even though the cell contents of the key components, Ubc9 and PML, did not increase after UAE inhibition.

SUMOylated proteins also accumulate in PML-NB during heat shock (shift from 37 to 43 °C) (35). However, in contrast to inhibition of UAE or proteasomes, this accumulation of SUMO-modified proteins becomes evident by only 2 h after a shift from 37 to 43 °C and is not inhibited by CHX (Fig. 1*H*). Also, most of the SUMOylated proteins reported to increase or decrease after heat shock differed from those found to change in levels after TAK243 treatment, but a third were identical. By contrast, proteasome inhibition and blocking ubiquitination affected the levels of SUMOylated proteins very similarly (84–90% overlap) (Tables S3 and S4), as would be expected if in both cases these changes in SUMOylated species resulted from a blockage of the UPS. Unlike these conditions, which prevent degradation by this pathway, upon heat shock, overall protein degradation by the UPS is about 2-fold greater than at 37 °C (68) through increased ubiquitination of thermally damaged proteins (68). The rise and fall of SUMOylated proteins following heat shock also differs from the response to TAK243 and MG132 in that it is not blocked by CHX and becomes evident sooner (35). Blocking proteolysis by the UPS causes a buildup of short-lived (*e.g.* misfolded or regulatory) proteins that are all newly synthesized, and these species seem to rise or fall most in the nucleus. By contrast, heat shock triggers rapid degradation of pre-existent relatively stable proteins that presumably are thermally damaged at 43 °C.^5^ Presumably, the large increase in levels of ubiquitinated substrates after heat shock exceeds the degradative capacity of the nuclei and leads to an accumulation in SUMOylated form. Thus, these very different types of cellular stress have some common mechanisms, but the heat shock clearly affects an additional set of nuclear proteins that perhaps are temperature-sensitive.

Because many secreted and membrane proteins are degraded through the ERAD pathway, not surprisingly, TAK243 treatment elicits the unfolded protein response (38). In fact, SUMOylated species of all transcription factors catalyzing this stress response rose dramatically following UAE inhibition. These various observations are all related to protein quality control in the nucleus and possible involvement of SUMO in this process (69). A number of observations (27) have suggested that PML-NBs function as important centers for protein quality control in the nucleus, perhaps to isolate potentially toxic undegraded nuclear proteins such as SUMO2/3 conjugates by phase separation before ubiquitination. Such a mechanism appears analogous to the isolation of nondegraded ubiquitinated proteins in cytosolic aggresomes by p62 (40) and would predict a greatly enlarged role for PML and SUMOylation upon heat shock or after inhibition of the UPS, as we described here.

## Experimental procedures

### Cell lines and growth conditions

Neuroblastoma cells M17 (ATCC, CRL-2267) and SH-SY5Y cells (CRL-2266) were cultured in DMEM/F-12 (1:1) media (Mediatech, 10-092-CV). HEK293A cells, HeLa cells, and U2OS cells were cultured in DMEM (Life Technologies, Inc., 41966-052). HeLa cells and U2OS cells stably expressing His_10_–SUMO2 were described previously (70, 71) and were cultured in DMEM. All media contained 10% fetal bovine serum (Sigma, F6178, 100 ml) and 1% penicillin/streptomycin solution (Life Technologies, Inc., 15070-063). All cells were maintained in a humidified incubator at 37 °C and 5% CO_2_.

### Transient knockdown of Ubc9, PML, or RNF4 by siRNA

siRNAs for Ubc9 (L-004910-00-0005), PML (L-006547-00-0005), or RNF4 (L-006557-00-0005) were purchased from GE Healthcare-Dharmacon. To knock down genes in HEK293A or HeLa cells (parental or His_10_–SUMO2 cells), the transfection mixture contained 20 pmol of siRNA and 1 μl of Lipofectamine 2000, which was prepared in 100 μl of Opti-MEM® and mixed at room temperature for 20 min, before addition to the cells (cultured with 500 μl of penicillin/streptomycin-free DMEM in a 24-well plate until 30–40% confluency). As a control, we prepared a mixture with only the transfection reagent and no siRNA.

### Production of lentivirus

To produce lentivirus, plasmids for lentiviral production and target shRNA as well as the control shRNA SHC002 were derived from the Mission shRNA library (Sigma). The Sigma catalogue numbers of these plasmids are TRCN0000004145 (PIAS1) and TRCN0000230128 (PIAS2); 293T cells were transfected at 60% confluency in a 15-cm dish with 13.7 μg of shRNA-encoding plasmid, helper plasmids pCMV-VSVG (7.5 μg), pMDLg-RRE (11.4 μg), pRSV-REV (5.4 μg), and 114 μg polyethyleneimine. Viral particles were purified by filtration with 0.45-μm filters.

### Knockdown of PIAS1, -2, -3, and -4 using lentiviral particle transduction

For shRNA-mediated knockdown experiments, 2 × 10^5^ cells seeded in a 6-well format were transduced with lentiviral particles expressing shRNAs targeting PIAS1, -2, -3, and -4. Transduction was performed with a multiplicity of infection of 3 in DMEM complete media containing 1 μg/ml Polybrene. 24 h after infection the medium was replaced. Cells were lysed 3 days after infection in SDS-Nonidet P-40 TBS lysis buffer (SNTBS) (containing 2% SDS, 1% Nonidet P-40, 150 mm NaCl, and 50 mm Tris-Cl, pH 7.0) for further analysis.

### Treatment of cells with UAE inhibitors and other compounds

Stock solutions were prepared for the following inhibitors: MG132 (Boston Biochem, I-130, 10 mm, DMSO); bortezomib (LC Laboratories, B-1408, 100 mm, DMSO); TAK243 (Active Biochem, A1384, 10 mm, DMSO); ML4924 (Boston Biochem, I-502, 20 mm, DMSO). The SAE inhibitor ML-792 (20 mm, DMSO) was kindly provided by Takeda Co.

### Immunostaining

HeLa cells (parental cells or cells expressing His_10_–SUMO2) were seeded in Lab-TekII 8-well chamber slides (Thermo Fisher Scientific, 154534). After treatment with TAK243, ML-792, or CHX, the cells were fixed with 4% paraformaldehyde for 15 min at room temperature, permeabilized with 1% Triton X-100 for 15 min at room temperature, and then rinsed twice with PBST (PBS with 0.05% Tween 20). Immunostaining was performed with anti-SUMO2/3 antibody (Abcam, ab-81371 (8A2), 1:100) or anti-PML antibody (Bethyl, A301-167A, 1:100). The secondary antibodies were Alexa Fluor 488-conjugated anti-mouse secondary antibody (Life Technologies, Inc., A-11001, 1:500) and Alexa Fluor 555-conjugated anti-rabbit secondary antibody (Life Technologies, Inc., A-21428, 1:500). Nuclei were counter-stained with DAPI in the mounting media (Life Technologies, Inc., P36966). Images were taken with a Nikon Lucille spinning-disk confocal microscope equipped with a Hamamatsu ORCA-ER–cooled CCD camera (confocal, for 488- and 555-nm channels, and widefield, for the DAPI channel) and a ×100/1.4 oil objective at room temperature, remotely controlled with MetaMorph image acquisition software.

### Lysate preparation and fractionation

Cells were lysed in room temperature SNTBS containing 2% SDS, 1% Nonidet P-40, 150 mm NaCl, and 50 mm Tris-Cl, pH 7.0, and sonicated. Alternatively, proteins were fractionated based on their ability to remain in the soluble supernatant after centrifugation (10,000 × *g* for 10 min) in mild detergent. For this purpose, cells were lysed in ice-cold 1% Triton X-100 lysis buffer (50 mm Tris-Cl, pH 7.4, 150 mm NaCl, 1 mm NaF, 1 mm EDTA, 1 mm Na_3_VO_4_, 1 mm dithiothreitol (DTT), 1% Triton X-100, protease inhibitor mixture tablet (Roche Applied Science)) for 20 min, and centrifuged (10,000 × *g* for 10 min). The pellets were solubilized in 2% SDS, 50 mm Tris-Cl, pH 7.4, and sonicated.

### Subcellular fractionation

To fractionate proteins into cytoplasmic, nuclear, and chromatin-bound fractions, cells were grown in 6-cm dishes, scraped with PBS, and centrifuged (1000 rpm for 2 min). Input sample was lysed in SNTBS. For fractionation purposes, a separate sample of cells was lysed with buffer containing 10 mm HEPES, 10 mm KCl, 1.5 mm MgCl_2_, 10% glycerol, 340 mm saccharose, 1 mm DTT, protease inhibitor mixture tablet (Roche Applied Science), 0.1% Triton for 8 min on ice. After centrifugation (13,700 rpm for 5 min), the supernatant was taken as the cytosolic fraction. To obtain the nuclear fraction, the pellet from the previous centrifugation step was lysed in buffer containing 3 mm EDTA, 200 μm EGTA, 1 mm DTT, protease inhibitor mixture tablet (Roche Applied Science). After centrifugation (4000 rpm for 4 min), the supernatant was taken as the nuclear fraction, and the pellet was lysed in SNTBS buffer containing additional 3 mm EDTA, 200 μm EGTA, 1 mm DTT, protease inhibitor mixture tablet (Roche Applied Science), and taken as the chromatin-bound fraction. Equal amounts of protein from each fraction, including the input lysate, were subsequently separated by SDS-PAGE and analyzed by Western blotting.

### Western blotting

To detect proteins, we used the following antibodies: against GAPDH (Sigma, G8795, 200 μl, 1:10,000); Ub (P4D1, Santa Cruz Biotechnology, sc-8017, 1:2000); Lys-48–specific poly-Ub (Cell Signaling Technology, 8081S, 1:2000); SUMO2/3 (Abcam, ab-3742, 1:2000, or ab-81371 (8A2), 1:2000); SUMO1 (Invitrogen, 332400, 1:500); lamin (Santa Cruz Biotechnology, sc-20682, 1:10000); His (Roche Applied Science, 11922416001, 1:5000); Ubc9 (Abgent, AM1261a-ev, 1:1000); PML (Bethyl, A301-167A, 1:1000); RNF4 (R&D Scientific, AF7964-SP, 1:1000); SENP1 (Abcam, ab108981, 1:1000); SENP7 (Bethyl, A302-270A, 1:1000); c-Myc (Y69, Abcam, ab32072, 1:1000); β-tubulin (Cell Signaling Technology, 2128S); histone H4 (Abcam, ab10158/100); PIAS1 (D33A7) (Cell Signaling Technology, 3550S, 1:500); PIAS2 (Abcam, ab126601, 1:500); PIAS3 (Cell Signaling Technology, 9042S, 1:500); PIAS4 (Cell Signaling Technology, 4392S, 1:500); CDCA7L (Bethyl, A300/846A, 1:1000); and HIF1α (BD Transduction Laboratories, 610959, 1:1000). Antibodies for Ufm1 (Abcam, ab109305, 1:1000), Isg15 (Cell Signaling Technology, 2743, 1:1000), and Nedd8 (Abcam, ab81264, 1:1000) were kindly provided by Katharina Witting. To detect proteins via enhanced chemiluminescence, we used horseradish peroxidase–conjugated goat anti-mouse (Promega, W4021, 1:10,000) or goat anti-rabbit (Promega, W4011, 1:10,000) secondary antibodies and SuperSignal West Pico chemiluminescent substrate (Thermo Fisher Scientific, 34080). To detect via the Odyssey® CLx IR Imaging System, we used IRDye® 680LT goat anti-mouse IgG (H+L) (LiCor, 926-68020, 1:10,000), IRDye® 800CW donkey anti-rabbit IgG (H+L) (LiCor, 926-32213, 1:10,000), or IRDye® 800CW donkey anti-goat IgG (H+L) (LiCor, 925-32214, 1:10,000) secondary antibodies. Quantification of signals by densitometry was performed using the Odyssey® CLx IR Imaging System. Actin, GAPDH, or total protein (measured by Ponceau S staining) was used as the loading control for cell lysate soluble in 1% Triton X-100, and lamin was used as the loading control for the pellet fraction.

### Purification of His_10_–SUMO2-conjugated proteins

The purification of His_10_–SUMO2-conjugated proteins from HeLa cells or U2OS cells was performed as described previously (46, 70, 71). Cells were washed, scraped, and collected in ice-cold PBS. For total lysates, a small aliquot of cells was kept separately and lysed in 2% SDS, 50 mm Tris-Cl, pH 7.4. For His_10_–SUMO2 purification, cell pellets from three 15-cm plates were lysed in 6 ml of 6 m guanidine-HCl, pH 8.0 (6 m guanidine-HCl, 0.1 m Na_2_HPO_4_/NaH_2_PO_4_, 10 mm Tris, pH 8.0), snap-frozen using liquid nitrogen, thawed at room temperature, and sonicated for 5 s at 30 watts for four bursts. The same amount of total protein was subjected to pulldown using 90 μl of Ni-NTA–agarose beads (Qiagen, 30210) in the presence of 50 mm imidazole and 5 mm β-mercaptoethanol. After binding, the Ni-NTA–agarose was washed using 900 μl of wash buffers A–D. (Wash buffer A: 6 m guanidine-HCl, 0.1 m Na_2_HPO_4_/NaH_2_PO_4_, pH 8.0, 10 mm Tris-Cl, pH 8.0, 10 mm imidazole, 5 mm β-mercaptoethanol, 0.1% Triton X-100; Wash buffer B: 8 m urea, 0.1 m Na_2_HPO_4_/NaH_2_PO_4_, pH 8.0, 10 mm Tris-Cl, pH 8.0, 10 mm imidazole, pH 8.0, 5 mm β-mercaptoethanol, 0.1% Triton X-100; Wash buffer C: 8 m urea, 0.1 m Na_2_HPO_4_/NaH_2_PO_4_, pH 6.3, 10 mm Tris-Cl, pH 6.3, 10 mm imidazole, pH 7.0, 5 mm β-mercaptoethanol; and Wash buffer D: 8 m urea, 0.1 m Na_2_HPO_4_/NaH_2_PO_4_, pH 6.3, 10 mm Tris-Cl, pH 6.3, 5 mm β-mercaptoethanol.) Samples were eluted in 270 μl of elution buffer (7 m urea, 0.1 m NaH_2_PO_4_/Na_2_HPO_4_, 10 mm Tris-Cl, pH 7.0, and 500 mm imidazole, pH 7.0).

### Mass spectrometry

LC-MS/MS analysis was performed on an EASY-nLC 1000 system (Proxeon, Odense, Denmark) connected to a Q-Exactive Orbitrap (Thermo Fisher Scientific, Germany) through a nano-electrospray ion source. The Q-Exactive was coupled to a 15-cm analytical column with an inner diameter of 75 μm, packed in-house with 1.9 μm C18-AQ beads (Reprospher-DE, Pur, Dr. Maish, Ammerbuch-Entringen, Germany).

The chromatography gradient length was 120 min from 2 to 95% acetonitrile in 0.1% formic acid at a flow rate of 200 nl/min. The mass spectrometer was operated in data-dependent acquisition mode with a top-10 method. Full-scan MS spectra were acquired at a target value of 3 × 10^6^ and a resolution of 70,000, and the higher-collisional dissociation tandem mass spectra (MS/MS) were recorded at a target value of 1 × 10^5^ and with a resolution of 17,500 with a normalized collision energy of 25%. The maximum MS1 and MS2 injection times were 20 and 60 ms, respectively. The precursor ion masses of scanned ions were dynamically excluded from MS/MS analysis for 60 s. Ions with charge 1, and greater than 6, were excluded from triggering MS2 analysis.

### Mass spectrometry data analysis

Proteomics data were analyzed using MaxQuant software (version 1.5.5.1) according to Ref. 72 using default settings with the following modifications: maximum number of mis-cleavages by trypsin was set to 3. LFQ was employed with the Fast-LFQ algorithm disabled. We performed the search against an *in silico*-digested UniProt reference proteome for *Homo sapiens* (Sept. 7, 2016). Maximum peptide mass was set to 5000 Da. The match-between-runs feature was enabled with a 0.7-min match time window and a 20-min alignment time window.

The analysis output from MaxQuant was further processed in the Perseus (version 1.5.5.3) computational platform (73). Proteins identified as common contaminants, only identified by site or reverse peptide, were filtered out, and then all the LFQ intensities were log2 transformed. Different biological repeats of the experiment were grouped, and only protein groups identified in all four biological replicates in at least one group were included for further analysis. Missing values were imputed using normally distributed values with a 1.8 downshift (log2) and a randomized 0.3 width (log2) considering whole-matrix values in Perseus. Z-scores were calculated, and a heatmap was generated. ANOVAs and *t* tests were performed with a permutation-based false discovery rate of 0.05 to test for differences between groups. Protein groups with an average LFQ value in the His-SUMO samples that were higher than in the parental control samples and a statistical significant difference by ANOVA were considered to be SUMO targets. Spreadsheets from the statistical analysis output were further processed in Microsoft Excel for comprehensive visualization and data analysis.

String analysis and visualization of the proteomics data results were performed using Cytoscape version 3.7.0 with plug-ins: StringApp 1.4.0 and MCode 1.5.1. For the string analysis the confidence cutoff was set to 0.4. Gene Ontology analysis was performed with the PANTHER over-representation test released on Nov. 13, 2018, version 13.1, from the Gene Ontology consortium.

### Mass spectrometry data availability

The MS proteomics data have been deposited to the ProteomeXchange Consortium via the PRIDE (74) partner repository with the dataset identifier PXD011852.

### Statistics

For the statistics assay for Fig. 3 and Fig. S3, we used nonparametric Kruskal-Wallis tests. Asterisk indicates that the *p* value is smaller than 0.05.

## Author contributions

Z. S., A. C. O. V., and A. L. G. conceptualization; Z. S., T. B., and R. G.-P. data curation; Z. S., T. B., R. G.-P., A. C. O. V., and A. L. G. formal analysis; Z. S., T. B., and R. G.-P. validation; Z. S., T. B., and R. G.-P. investigation; Z. S., T. B., R. G.-P., and A. L. G. visualization; Z. S., T. B., R. G.-P., and A. L. G. methodology; Z. S., A. C. O. V., and A. L. G. writing-original draft; Z. S., A. C. O. V., and A. L. G. writing-review and editing; R. G.-P. software; A. C. O. V. and A. L. G. funding acquisition; A. C. O. V. and A. L. G. project administration; A. L. G. resources; A. L. G. supervision.

## Footnotes

5 J. Zhao and A. L. Goldberg, unpublished observations.

## Footnotes

4 The abbreviations used are:

UPS: ubiquitin–proteasome system
Ub: ubiquitin
SUMO: small ubiquitin-related modifier
SCM: SUMOylation consensus motif
PML: promyelocytic leukemia protein
PML-NB: PML-nuclear body
SIM: SUMO-interaction motif
DMEM: Dulbecco's modified Eagle's medium
GAPDH: glyceraldehyde-3-phosphate dehydrogenase
CHX: cycloheximide
ER: endoplasmic reticulum
Ni-NTA: nickel-nitrilotriacetic acid
STUbL: SUMO-targeted Ub ligase
BTZ: bortezomib
UAE: Ub-activating enzyme
SAE: SUMO-activating enzyme
LFQ: label-free quantification
PARP: poly(ADP-ribose) polymerase
MTS: 3-(4,5-dimethylthiazol-2-yl)-5-(3-carboxymethoxyphenyl)-2-(4-sulfophenyl)-2H-tetrazolium
SENP: sentrin-specific protease
ERAD: endoplasmic reticulum-associated degradation
TRIM: tripartite motif
DAPI: 4,6-diamidino-2-phenylindole
ANOVA: analysis of variance.

## References

[1] Hochstrasser M. (2009) Origin and function of ubiquitin-like proteins. Nature 458, 422–429 10.1038/nature07958 19325621 PMC2819001

[2] Zhao J., Zhai B., Gygi S. P., and Goldberg A. L. (2015) mTOR inhibition activates overall protein degradation by the ubiquitin proteasome system as well as by autophagy. Proc. Natl. Acad. Sci. U.S.A. 112, 15790–15797 10.1073/pnas.1521919112 26669439 PMC4703015

[3] Finley D. (2009) Recognition and processing of ubiquitin-protein conjugates by the proteasome. Annu. Rev. Biochem. 78, 477–513 10.1146/annurev.biochem.78.081507.101607 19489727 PMC3431160

[4] Collins G. A., and Goldberg A. L. (2017) The logic of the 26S proteasome. Cell 169, 792–806 10.1016/j.cell.2017.04.023 28525752 PMC5609836

[5] van der Veen A. G., and Ploegh H. L. (2012) Ubiquitin-like proteins. Annu. Rev. Biochem. 81, 323–357 10.1146/annurev-biochem-093010-153308 22404627

[6] Hendriks I. A., and Vertegaal A. C. (2016) A comprehensive compilation of SUMO proteomics. Nat. Rev. Mol. Cell Biol. 17, 581–595 10.1038/nrm.2016.81 27435506

[7] Gareau J. R., and Lima C. D. (2010) The SUMO pathway: emerging mechanisms that shape specificity, conjugation and recognition. Nat. Rev. Mol. Cell Biol. 11, 861–871 10.1038/nrm3011 21102611 PMC3079294

[8] Wilkinson K. A., and Henley J. M. (2010) Mechanisms, regulation and consequences of protein SUMOylation. Biochem. J. 428, 133–145 10.1042/BJ20100158 20462400 PMC3310159

[9] Seeler J. S., and Dejean A. (2017) SUMO and the robustness of cancer. Nat. Rev. Cancer 17, 184–197 10.1038/nrc.2016.143 28134258

[10] Tatham M. H., Jaffray E., Vaughan O. A., Desterro J. M., Botting C. H., Naismith J. H., and Hay R. T. (2001) Polymeric chains of SUMO-2 and SUMO-3 are conjugated to protein substrates by SAE1/SAE2 and Ubc9. J. Biol. Chem. 276, 35368–35374 10.1074/jbc.M104214200 11451954

[11] Bernier-Villamor V., Sampson D. A., Matunis M. J., and Lima C. D. (2002) Structural basis for E2-mediated SUMO conjugation revealed by a complex between ubiquitin-conjugating enzyme Ubc9 and RanGAP1. Cell 108, 345–356 10.1016/S0092-8674(02)00630-X 11853669

[12] Hickey C. M., Wilson N. R., and Hochstrasser M. (2012) Function and regulation of SUMO proteases. Nat. Rev. Mol. Cell Biol. 13, 755–766 10.1038/nrm3478 23175280 PMC3668692

[13] Flotho A., and Melchior F. (2013) Sumoylation: a regulatory protein modification in health and disease. Annu. Rev. Biochem. 82, 357–385 10.1146/annurev-biochem-061909-093311 23746258

[14] Psakhye I., and Jentsch S. (2012) Protein group modification and synergy in the SUMO pathway as exemplified in DNA repair. Cell 151, 807–820 10.1016/j.cell.2012.10.021 23122649

[15] Banani S. F., Rice A. M., Peeples W. B., Lin Y., Jain S., Parker R., and Rosen M. K. (2016) Compositional control of phase-separated cellular bodies. Cell 166, 651–663 10.1016/j.cell.2016.06.010 27374333 PMC4967043

[16] Lallemand-Breitenbach V., and de Thé H. (2018) PML nuclear bodies: from architecture to function. Curr. Opin. Cell Biol. 52, 154–161 10.1016/j.ceb.2018.03.011 29723661

[17] Ablain J., Rice K., Soilihi H., de Reynies A., Minucci S., and de Thé H. (2014) Activation of a promyelocytic leukemia–tumor protein 53 axis underlies acute promyelocytic leukemia cure. Nat. Med. 20, 167–174 10.1038/nm.3441 24412926

[18] de Thé H., Pandolfi P. P., and Chen Z. (2017) Acute promyelocytic leukemia: a paradigm for oncoprotein-targeted cure. Cancer Cell 32, 552–560 10.1016/j.ccell.2017.10.002 29136503

[19] Sahin U., Ferhi O., Jeanne M., Benhenda S., Berthier C., Jollivet F., Niwa-Kawakita M., Faklaris O., Setterblad N., de Thé H., and Lallemand-Breitenbach V. (2014) Oxidative stress-induced assembly of PML nuclear bodies controls sumoylation of partner proteins. J. Cell Biol. 204, 931–945 10.1083/jcb.201305148 24637324 PMC3998805

[20] Lomonte P. (2016) The interaction between herpes simplex virus 1 genome and promyelocytic leukemia nuclear bodies (PML-NBs) as a hallmark of the entry in latency. Microb. Cell 3, 569–572 10.15698/mic2016.11.541 28357326 PMC5349213

[21] Bailey D., and O'Hare P. (2005) Comparison of the SUMO1 and ubiquitin conjugation pathways during the inhibition of proteasome activity with evidence of SUMO1 recycling. Biochem. J. 392, 271–281 10.1042/BJ20050873 16117725 PMC1316262

[22] Hsu K. S., and Kao H. Y. (2018) PML: regulation and multifaceted function beyond tumor suppression. Cell Biosci. 8, 5 10.1186/s13578-018-0204-8 29416846 PMC5785837

[23] Ferbeyre G., de Stanchina E., Querido E., Baptiste N., Prives C., and Lowe S. W. (2000) PML is induced by oncogenic ras and promotes premature senescence. Genes Dev. 14, 2015–2027 10950866 PMC316863

[24] Sahin U., Lallemand-Breitenbach V., and de Thé H. (2014) PML nuclear bodies: regulation, function and therapeutic perspectives. J. Pathol. 234, 289–291 10.1002/path.4426 25138686

[25] Wang Z. G., Ruggero D., Ronchetti S., Zhong S., Gaboli M., Rivi R., and Pandolfi P. P. (1998) PML is essential for multiple apoptotic pathways. Nat. Genet. 20, 266–272 10.1038/3073 9806545

[26] Shen T. H., Lin H. K., Scaglioni P. P., Yung T. M., and Pandolfi P. P. (2006) The mechanisms of PML-nuclear body formation. Mol. Cell 24, 331–339 10.1016/j.molcel.2006.09.013 17081985 PMC1978182

[27] Guo L., Giasson B. I., Glavis-Bloom A., Brewer M. D., Shorter J., Gitler A. D., and Yang X. (2014) A cellular system that degrades misfolded proteins and protects against neurodegeneration. Mol. Cell 55, 15–30 10.1016/j.molcel.2014.04.030 24882209 PMC4445634

[28] Hendriks I. A., D'Souza R. C., Yang B., Verlaan-de Vries M., Mann M., and Vertegaal A. C. (2014) Uncovering global SUMOylation signaling networks in a site-specific manner. Nat. Struct. Mol. Biol. 21, 927–936 10.1038/nsmb.2890 25218447 PMC4259010

[29] Zilio N., Eifler-Olivi K., and Ulrich H. D. (2017) Functions of SUMO in the maintenance of genome stability. Adv. Exp. Med. Biol. 963, 51–87 10.1007/978-3-319-50044-7_4 28197906

[30] Desterro J. M., Rodriguez M. S., and Hay R. T. (1998) SUMO-1 modification of IκBα inhibits NF-κB activation. Mol. Cell 2, 233–239 10.1016/S1097-2765(00)80133-1 9734360

[31] Rott R., Szargel R., Shani V., Hamza H., Savyon M., Abd Elghani F., Bandopadhyay R., and Engelender S. (2017) SUMOylation and ubiquitination reciprocally regulate α-synuclein degradation and pathological aggregation. Proc. Natl. Acad. Sci. U.S.A. 114, 13176–13181 10.1073/pnas.1704351114 29180403 PMC5740625

[32] Tatham M. H., Geoffroy M. C., Shen L., Plechanovova A., Hattersley N., Jaffray E. G., Palvimo J. J., and Hay R. T. (2008) RNF4 is a poly-SUMO-specific E3 ubiquitin ligase required for arsenic-induced PML degradation. Nat. Cell Biol. 10, 538–546 10.1038/ncb1716 18408734

[33] Yin Y., Seifert A., Chua J. S., Maure J. F., Golebiowski F., and Hay R. T. (2012) SUMO-targeted ubiquitin E3 ligase RNF4 is required for the response of human cells to DNA damage. Genes Dev. 26, 1196–1208 10.1101/gad.189274.112 22661230 PMC3371408

[34] Galanty Y., Belotserkovskaya R., Coates J., and Jackson S. P. (2012) RNF4, a SUMO-targeted ubiquitin E3 ligase, promotes DNA double-strand break repair. Genes Dev. 26, 1179–1195 10.1101/gad.188284.112 22661229 PMC3371407

[35] Tatham M. H., Matic I., Mann M., and Hay R. T. (2011) Comparative proteomic analysis identifies a role for SUMO in protein quality control. Sci. Signal. 4, rs4 10.1126/scisignal.2001484 21693764

[36] Golebiowski F., Matic I., Tatham M. H., Cole C., Yin Y., Nakamura A., Cox J., Barton G. J., Mann M., and Hay R. T. (2009) System-wide changes to SUMO modifications in response to heat shock. Sci. Signal. 2, ra24 10.1126/scisignal.2000282 19471022

[37] Barghout S. H., Patel P. S., Wang X., Xu G. W., Kavanagh S., Halgas O., Zarabi S. F., Gronda M., Hurren R., Jeyaraju D. V., MacLean N., Brennan S., Hyer M. L., Berger A., Traore T., et al (2019) Preclinical evaluation of the selective small-molecule UBA1 inhibitor, TAK-243, in acute myeloid leukemia. Leukemia 33, 37–51 10.1038/s41375-018-0167-0 29884901

[38] Hyer M. L., Milhollen M. A., Ciavarri J., Fleming P., Traore T., Sappal D., Huck J., Shi J., Gavin J., Brownell J., Yang Y., Stringer B., Griffin R., Bruzzese F., Soucy T., et al (2018) A small-molecule inhibitor of the ubiquitin activating enzyme for cancer treatment. Nat. Med. 24, 186–193 10.1038/nm.4474 29334375

[39] He X., Riceberg J., Soucy T., Koenig E., Minissale J., Gallery M., Bernard H., Yang X., Liao H., Rabino C., Shah P., Xega K., Yan Z. H., Sintchak M., Bradley J., et al (2017) Probing the roles of SUMOylation in cancer cell biology by using a selective SAE inhibitor. Nat. Chem. Biol. 13, 1164–1171 10.1038/nchembio.2463 28892090

[40] Sha Z., Schnell H. M., Ruoff K., and Goldberg A. (2018) Rapid induction of p62 and GABARAPL1 upon proteasome inhibition promotes survival before autophagy activation. J. Cell Biol. 217, 1757–1776 10.1083/jcb.201708168 29535191 PMC5940303

[41] Maghames C. M., Lobato-Gil S., Perrin A., Trauchessec H., Rodriguez M. S., Urbach S., Marin P., and Xirodimas D. P. (2018) NEDDylation promotes nuclear protein aggregation and protects the ubiquitin proteasome system upon proteotoxic stress. Nat. Commun. 9, 4376 10.1038/s41467-018-06365-0 30349034 PMC6197266

[42] Dundr M. (2012) Nuclear bodies: multifunctional companions of the genome. Curr. Opin. Cell Biol. 24, 415–422 10.1016/j.ceb.2012.03.010 22541757 PMC3372688

[43] Wang J., Shiels C., Sasieni P., Wu P. J., Islam S. A., Freemont P. S., and Sheer D. (2004) Promyelocytic leukemia nuclear bodies associate with transcriptionally active genomic regions. J. Cell Biol. 164, 515–526 10.1083/jcb.200305142 14970191 PMC2171989

[44] Tahk S., Liu B., Chernishof V., Wong K. A., Wu H., and Shuai K. (2007) Control of specificity and magnitude of NF-κB and STAT1-mediated gene activation through PIASy and PIAS1 cooperation. Proc. Natl. Acad. Sci. U.S.A. 104, 11643–11648 10.1073/pnas.0701877104 17606919 PMC1913887

[45] Erker Y., Neyret-Kahn H., Seeler J. S., Dejean A., Atfi A., and Levy L. (2013) Arkadia, a novel SUMO-targeted ubiquitin ligase involved in PML degradation. Mol. Cell. Biol. 33, 2163–2177 10.1128/MCB.01019-12 23530056 PMC3648077

[46] Hendriks I. A., and Vertegaal A. C. (2016) Label-free identification and quantification of SUMO target proteins. Methods Mol. Biol. 1475, 171–193 10.1007/978-1-4939-6358-4_13 27631806

[47] Gene Ontology Consortium. (2015) Gene Ontology Consortium: going forward. Nucleic Acids Res. 43, D1049–D1056 10.1093/nar/gku1179 25428369 PMC4383973

[48] Hendriks I. A., Lyon D., Young C., Jensen L. J., Vertegaal A. C., and Nielsen M. L. (2017) Site-specific mapping of the human SUMO proteome reveals co-modification with phosphorylation. Nat. Struct. Mol. Biol. 24, 325–336 10.1038/nsmb.3366 28112733

[49] Kumar R., González-Prieto R., Xiao Z., Verlaan-de Vries M., and Vertegaal A. C. O. (2017) The STUbL RNF4 regulates protein group SUMOylation by targeting the SUMO conjugation machinery. Nat. Commun. 8, 1809 10.1038/s41467-017-01900-x 29180619 PMC5703878

[50] Hou X., Yang Z., Zhang K., Fang D., and Sun F. (2017) SUMOylation represses the transcriptional activity of the unfolded protein response transducer ATF6. Biochem. Biophys. Res. Commun. 494, 446–451 10.1016/j.bbrc.2017.10.103 29061306 PMC5698096

[51] Hong Y., Rogers R., Matunis M. J., Mayhew C. N., Goodson M. L., Park-Sarge O. K., Sarge K. D., and Goodson M. (2001) Regulation of heat shock transcription factor 1 by stress-induced SUMO-1 modification. J. Biol. Chem. 276, 40263–40267 10.1074/jbc.M104714200 11514557

[52] Goodson M. L., Hong Y., Rogers R., Matunis M. J., Park-Sarge O. K., and Sarge K. D. (2001) Sumo-1 modification regulates the DNA binding activity of heat shock transcription factor 2, a promyelocytic leukemia nuclear body associated transcription factor. J. Biol. Chem. 276, 18513–18518 10.1074/jbc.M008066200 11278381

[53] Imoto S., Ohbayashi N., Ikeda O., Kamitani S., Muromoto R., Sekine Y., and Matsuda T. (2008) Sumoylation of Smad3 stimulates its nuclear export during PIASy-mediated suppression of TGF-β signaling. Biochem. Biophys. Res. Commun. 370, 359–365 10.1016/j.bbrc.2008.03.116 18384750

[54] Lin X., Liang M., Liang Y. Y., Brunicardi F. C., and Feng X. H. (2003) SUMO-1/Ubc9 promotes nuclear accumulation and metabolic stability of tumor suppressor Smad4. J. Biol. Chem. 278, 31043–31048 10.1074/jbc.C300112200 12813045

[55] Long J., Wang G., He D., and Liu F. (2004) Repression of Smad4 transcriptional activity by SUMO modification. Biochem. J. 379, 23–29 10.1042/bj20031867 14750902 PMC1224064

[56] Myatt S. S., Kongsema M., Man C. W., Kelly D. J., Gomes A. R., Khongkow P., Karunarathna U., Zona S., Langer J. K., Dunsby C. W., Coombes R. C., French P. M., Brosens J. J., and Lam E. W. (2014) SUMOylation inhibits FOXM1 activity and delays mitotic transition. Oncogene 33, 4316–4329 10.1038/onc.2013.546 24362530 PMC4096495

[57] Chen H., and Qi L. (2010) SUMO modification regulates the transcriptional activity of XBP1. Biochem. J. 429, 95–102 10.1042/BJ20100193 20408817 PMC2964647

[58] Schimmel J., Eifler K., Sigurethsson J. O., Cuijpers S. A., Hendriks I. A., Verlaan-de Vries M., Kelstrup C. D., Francavilla C., Medema R. H., Olsen J. V., and Vertegaal A. C. (2014) Uncovering SUMOylation dynamics during cell-cycle progression reveals FoxM1 as a key mitotic SUMO target protein. Mol. Cell 53, 1053–1066 10.1016/j.molcel.2014.02.001 24582501

[59] Rao H. B., Qiao H., Bhatt S. K., Bailey L. R., Tran H. D., Bourne S. L., Qiu W., Deshpande A., Sharma A. N., Beebout C. J., Pezza R. J., and Hunter N. (2017) A SUMO-ubiquitin relay recruits proteasomes to chromosome axes to regulate meiotic recombination. Science 355, 403–407 10.1126/science.aaf6407 28059716 PMC5569317

[60] Mukhopadhyay D., Ayaydin F., Kolli N., Tan S. H., Anan T., Kametaka A., Azuma Y., Wilkinson K. D., and Dasso M. (2006) SUSP1 antagonizes formation of highly SUMO2/3-conjugated species. J. Cell Biol. 174, 939–949 10.1083/jcb.200510103 17000875 PMC2064386

[61] Mimnaugh E. G., Chen H. Y., Davie J. R., Celis J. E., and Neckers L. (1997) Rapid deubiquitination of nucleosomal histones in human tumor cells caused by proteasome inhibitors and stress response inducers: effects on replication, transcription, translation, and the cellular stress response. Biochemistry 36, 14418–14429 10.1021/bi970998j 9398160

[62] Cappadocia L., Pichler A., and Lima C. D. (2015) Structural basis for catalytic activation by the human ZNF451 SUMO E3 ligase. Nat. Struct. Mol. Biol. 22, 968–975 10.1038/nsmb.3116 26524494 PMC4709122

[63] Weger S., Hammer E., and Heilbronn R. (2005) Topors acts as a SUMO-1 E3 ligase for p53 *in vitro* and *in vivo*. FEBS Lett. 579, 5007–5012 10.1016/j.febslet.2005.07.088 16122737

[64] Eisenhardt N., Chaugule V. K., Koidl S., Droescher M., Dogan E., Rettich J., Sutinen P., Imanishi S. Y., Hofmann K., Palvimo J. J., and Pichler A. (2015) A new vertebrate SUMO enzyme family reveals insights into SUMO-chain assembly. Nat. Struct. Mol. Biol. 22, 959–967 10.1038/nsmb.3114 26524493

[65] Chu Y., and Yang X. (2011) SUMO E3 ligase activity of TRIM proteins. Oncogene 30, 1108–1116 10.1038/onc.2010.462 20972456 PMC3103664

[66] Uversky V. N. (2017) Intrinsically disordered proteins in overcrowded milieu: membrane-less organelles, phase separation, and intrinsic disorder. Curr. Opin. Struct. Biol. 44, 18–30 10.1016/j.sbi.2016.10.015 27838525

[67] Reits E. A., Vos J. C., Grommé M., and Neefjes J. (2000) The major substrates for TAP *in vivo* are derived from newly-synthesized proteins. Nature 404, 774–778 10.1038/35008103 10783892

[68] Medicherla B., and Goldberg A. L. (2008) Heat shock and oxygen radicals stimulate ubiquitin-dependent degradation mainly of newly-synthesized proteins. J. Cell Biol. 182, 663–673 10.1083/jcb.200803022 18725537 PMC2518706

[69] Liebelt F., Sebastian R. M., Moore C. L., Mulder M. P. C., Ovaa H., Shoulders M. D., and Vertegaal A. C. O. (2019) SUMOylation and the HSF1-regulated chaperone network converge to promote proteostasis in response to heat shock. Cell Rep. 26, 236–249.e4 10.1016/j.celrep.2018.12.027 30605679 PMC6316133

[70] Xiao Z., Chang J. G., Hendriks I. A., Sigurðsson J. O., Olsen J. V., and Vertegaal A. C. (2015) System-wide analysis of SUMOylation dynamics in response to replication stress reveals novel small ubiquitin-like modified target proteins and acceptor lysines relevant for genome stability. Mol. Cell. Proteomics 14, 1419–1434 10.1074/mcp.O114.044792 25755297 PMC4424410

[71] Hendriks I. A., D'Souza R. C., Chang J. G., Mann M., and Vertegaal A. C. (2015) System-wide identification of wild-type SUMO-2 conjugation sites. Nat. Commun. 6, 7289 10.1038/ncomms8289 26073453 PMC4490555

[72] Tyanova S., Temu T., and Cox J. (2016) The MaxQuant computational platform for mass spectrometry-based shotgun proteomics. Nat. Protoc. 11, 2301–2319 10.1038/nprot.2016.136 27809316

[73] Tyanova S., Temu T., Sinitcyn P., Carlson A., Hein M. Y., Geiger T., Mann M., and Cox J. (2016) The Perseus computational platform for comprehensive analysis of (prote)omics data. Nat. Methods 13, 731–740 10.1038/nmeth.3901 27348712

[74] Vizcaíno J. A., Csordas A., del-Toro N., Dianes J. A., Griss J., Lavidas I., Mayer G., Perez-Riverol Y., Reisinger F., Ternent T., Xu Q. W., Wang R., and Hermjakob H. (2016) 2016 update of the PRIDE database and its related tools. Nucleic Acids Res. 44, D447–D456 10.1093/nar/gkv1145 26527722 PMC4702828

